# Surgery for chronic pancreatitis across Europe (ESCOPA): prospective multicentre study

**DOI:** 10.1093/bjs/znaf068

**Published:** 2025-04-29

**Authors:** Charlotte L van Veldhuisen, Charlotte A Leseman, Fleur E M De Rijk, Joana Marques-Antunes, Fabio Ausania, Orlin Belyaev, Frederik Berrevoet, Marja A Boermeester, Ugo Boggi, Stefan A Bouwense, Marco J Bruno, Olivier R Busch, Kevin C Conlon, Safi Dokmak, Massimo Falconi, Poya Ghorbani, Filip Gryspeerdt, Roel Haen, Arturan Ibrahimli, Jakob R Izbicki, Christina Krikke, Arto Kokkola, Lancelot Marique, J Sven D Mieog, Gennaro Nappo, Janis Pavulans, Haralds Plaudis, Geert Roeyen, Pasquale Scognamiglio, Domenico Tamburrino, Tore Tholfsen, Marie Toschka, Faik G Uzunoglu, Susan van Dieren, Casper H J Van Eijck, Jeanin E van Hooft, Hjalmar C van Santvoort, Robert C Verdonk, Rogier P Voermans, Anne Waage, Marc G Besselink

**Affiliations:** Department of Surgery, Amsterdam UMC, Location University of Amsterdam, Amsterdam, The Netherlands; Amsterdam Gastroenterology Endocrinology Metabolism, Amsterdam UMC, University of Amsterdam, Amsterdam, The Netherlands; Department of Research and Development, St Antonius Hospital, Nieuwegein, The Netherlands; Department of Surgery, Amsterdam UMC, Location University of Amsterdam, Amsterdam, The Netherlands; Amsterdam Gastroenterology Endocrinology Metabolism, Amsterdam UMC, University of Amsterdam, Amsterdam, The Netherlands; Amsterdam Gastroenterology Endocrinology Metabolism, Amsterdam UMC, University of Amsterdam, Amsterdam, The Netherlands; Department of Gastroenterology and Hepatology, Erasmus University Medical Centre, Rotterdam, The Netherlands; Department of Surgery, ULSEDV, Santa Maria da Feira, Portugal; Department of Hepatic, Pancreatic, Biliary, and Transplant Surgery, Clinic Hospital, University of Barcelona, IDIBAPS, Barcelona, Spain; Department of Surgery, St Josef-Hospital, Ruhr-University Bochum, Bochum, Germany; General and HPB Surgery and Liver Transplantations, Ghent University Hospital, Ghent, Belgium; Department of Surgery, Amsterdam UMC, Location University of Amsterdam, Amsterdam, The Netherlands; Amsterdam Gastroenterology Endocrinology Metabolism, Amsterdam UMC, University of Amsterdam, Amsterdam, The Netherlands; Division of General and Transplant Surgery, University of Pisa, Pisa, Italy; Department of Surgery, Maastricht University Medical Centre+, Maastricht, The Netherlands; Department of Gastroenterology and Hepatology, Erasmus University Medical Centre, Rotterdam, The Netherlands; Department of Surgery, Amsterdam UMC, Location University of Amsterdam, Amsterdam, The Netherlands; Amsterdam Gastroenterology Endocrinology Metabolism, Amsterdam UMC, University of Amsterdam, Amsterdam, The Netherlands; Department of Surgery, Trinity College Dublin, Tallaght Hospital, Dublin, Ireland; Department of HPB Surgery and Liver Transplantation, AP-HP, Beaujon Hospital, University of Paris Cité, Centre de Recherche sur l'Inflammation, INSERM Unité Mixte de Recherche 1149, Clichy, France; Pancreas Translational and Clinical Research Centre, IRCCS San Raffaele Scientific Institute, Università Vita-Salute, Milan, Italy; Department of Upper Abdominal Diseases, Karolinska University Hospital, Stockholm, Sweden; General and HPB Surgery and Liver Transplantations, Ghent University Hospital, Ghent, Belgium; Department of Surgery, Erasmus MC Cancer Institute, Rotterdam, The Netherlands; Department of Surgery, Liv Bona Dea Hospital, Baku, Azerbaijan; Department for General, Visceral and Thoracic Surgery, University Hospital Hamburg Eppendorf, Hamburg, Germany; Department of Surgery, University Medical Centre Groningen, University of Groningen, Groningen, The Netherlands; Department of Surgery, Helsinki University Hospital and University of Helsinki, Helsinki, Finland; Department of HPB Surgery and Liver Transplantation, AP-HP, Beaujon Hospital, University of Paris Cité, Centre de Recherche sur l'Inflammation, INSERM Unité Mixte de Recherche 1149, Clichy, France; Department of Surgery, Leiden University Medical Centre, Leiden, The Netherlands; Department of Biomedical Sciences, Humanitas University, Milan, Italy; Pancreatic Surgery Unit, Humanitas Clinical and Research Centre, IRCCS, Milan, Italy; Department of Surgery, Riga East Clinical University Hospital, Riga, Latvia; Department of Surgery, Riga East Clinical University Hospital, Riga, Latvia; Department of Hepatobiliary Transplantation and Endocrine Surgery, Antwerp University Hospital and University of Antwerp, Edegem, Belgium; Department for General, Visceral and Thoracic Surgery, University Hospital Hamburg Eppendorf, Hamburg, Germany; Pancreas Translational and Clinical Research Centre, IRCCS San Raffaele Scientific Institute, Università Vita-Salute, Milan, Italy; Department of Hepato-Pancreato-Biliary Surgery, Oslo University Hospital, Rikshospitalet, Oslo, Norway; Department of Surgery, St Josef-Hospital, Ruhr-University Bochum, Bochum, Germany; Department for General, Visceral and Thoracic Surgery, University Hospital Hamburg Eppendorf, Hamburg, Germany; Department of Surgery, Amsterdam UMC, Location University of Amsterdam, Amsterdam, The Netherlands; Amsterdam Gastroenterology Endocrinology Metabolism, Amsterdam UMC, University of Amsterdam, Amsterdam, The Netherlands; Department of Surgery, Erasmus MC Cancer Institute, Rotterdam, The Netherlands; Department of Gastroenterology and Hepatology, Leiden University Medical Centre, Leiden, The Netherlands; Department of Surgery, St Antonius Hospital, Nieuwegein, The Netherlands; Department of Surgery, University Medical Centre Utrecht, Utrecht, The Netherlands; Department of Gastroenterology and Hepatology, St Antonius Hospital, Nieuwegein, The Netherlands; Amsterdam Gastroenterology Endocrinology Metabolism, Amsterdam UMC, University of Amsterdam, Amsterdam, The Netherlands; Department of Gastroenterology and Hepatology, Amsterdam UMC, Amsterdam, The Netherlands; Department of Hepato-Pancreato-Biliary Surgery, Oslo University Hospital, Rikshospitalet, Oslo, Norway; Department of Surgery, Amsterdam UMC, Location University of Amsterdam, Amsterdam, The Netherlands; Amsterdam Gastroenterology Endocrinology Metabolism, Amsterdam UMC, University of Amsterdam, Amsterdam, The Netherlands

## Abstract

**Background:**

Randomized trials have demonstrated the superiority of surgery over endoscopy in patients with symptomatic chronic pancreatitis. However, large international studies quantifying the impact of surgery on chronic pancreatitis are lacking. The aim of this study was to evaluate current practice across Europe regarding indications, surgical techniques, and outcomes of surgery for chronic pancreatitis.

**Methods:**

A prospective multicentre study of consecutive patients undergoing surgery for symptomatic chronic pancreatitis from 22 centres in 13 countries from 1 June 2021 to 30 November 2022 was conducted. The outcome of interest in patients with pain as an indication was the Izbicki pain score at 6-month follow-up, with complete pain relief defined as an Izbicki pain score ≤10 and partial pain relief defined as an Izbicki pain score >10, but with a >50% decrease compared with the baseline score. Quality of life was assessed using Pancreatitis Quality of Life Instrument (PANQOLI) and 12-Item Short-Form (SF-12) surveys. Predictors of pain relief were analysed using multivariable analysis.

**Results:**

Overall, 207 patients underwent surgery (24.6% underwent surgical drainage procedures, 29.5% underwent duodenum-preserving head resections, and 45.9% underwent formal pancreatic resections). Before surgery, 48.8% used opioids and 51.2% had undergone prior endoscopic treatment. Major morbidity occurred in 14.0% and the 90-day mortality rate was 1.4%. Among 113 patients operated on for pain, the median Izbicki pain score decreased from 61.3 to 19.0 at 6 months (*P* < 0.001). Pain relief was achieved in 72.6% (43 patients reported complete pain relief and 39 patients reported partial pain relief). PANQOLI and SF-12 Physical Component Summary scores improved significantly (*P* < 0.001). Longer symptom duration (OR 0.95 (95% c.i. 0.90 to 1.00), *P* = 0.045) and use of opioids before surgery (OR 3.16 (95% c.i. 1.04 to 9.64), *P* = 0.043) predicted less pain relief.

**Conclusion:**

Surgery for chronic pancreatitis across Europe was performed with low morbidity. Patients reported good pain relief and improvements in quality-of-life scores. Multidisciplinary consultation is recommended for all patients with chronic pancreatitis before undergoing any intervention.

## Teaser

This prospective pan-European study found low morbidity and mortality rates, good pain relief, and improved quality of life after surgery for chronic pancreatitis and confirmed the need for earlier patient referral.

## Introduction

Patients with symptomatic chronic pancreatitis typically suffer from severe abdominal pain and recurrent pancreatitis flares^1,2^. These symptoms, often combined with considerable diagnostic and therapeutic delay, have a substantial impact on patient quality of life^3–5^. Timely surgery may provide satisfactory long-term functional outcomes, with no significant differences between various surgical procedures^6–8^. Based on several European randomized trials in patients with symptomatic chronic pancreatitis, the European HaPanEU guidelines advise surgery in patients with intractable pain from symptomatic chronic pancreatitis, suspected pancreatic cancer, and complications of adjacent organs^9–11^. However, patients are often still referred late to surgery for symptomatic chronic pancreatitis, only after months to years of previous unsuccessful medical and endoscopic treatment^3,12^.

Pancreatic morphology varies widely in patients with symptomatic chronic pancreatitis and thus surgical approaches may include surgical drainage procedures, duodenum-preserving pancreatic head resections (DPPHRs), and formal pancreatic resections. Recent international guidelines advise to tailor surgery based on morphological abnormalities (based on an enlarged/non-enlarged pancreatic head and a dilated/non-dilated pancreatic duct (PD))^1,13^. The use and outcomes of surgery for chronic pancreatitis across Europe are unclear, as international prospective multicentre studies are lacking. A prospective study should take baseline pain scores and baseline quality of life into account to assess the full impact of surgery.

The aim of this prospective pan-European study was to evaluate current practice across Europe regarding indications, surgical techniques, and outcomes of surgery for chronic pancreatitis, in terms of morbidity, 90-day mortality rate, pain relief, and quality of life, including baseline assessment.

## Methods

### Study design

This was a prospective multicentre observational study, coordinated by the Dutch Pancreatitis Study Group (DPSG) and endorsed by the Scientific and Research Committee of the European–African Hepato-Pancreato-Biliary Association (E-AHPBA). All member centres of the E-AHPBA were invited via e-mail to participate in this study. The study was conducted in accordance with the STROBE guidelines^14^ and was approved by the medical ethics committee of the Amsterdam UMC.

### Study participants

Consecutive patients with symptomatic chronic pancreatitis (for example pain, obstruction, or pancreatitis flares) undergoing any type of elective surgery for chronic pancreatitis in the participating centres between 1 June 2021 and 30 November 2022 were included. Outcomes were assessed during a 6-month follow-up interval. Patients were eligible for this study if they met the following inclusion criteria: adult patient with a diagnosis of symptomatic chronic pancreatitis according to the M-ANNHEIM criteria^15^; and consensus after Multidisciplinary Team discussion that surgery is indicated. Patients with suspected pancreatic cancer were excluded.

### Investigated outcomes

The outcome of interest was pain, assessed by the Izbicki pain score at baseline (before surgery) and 1, 3, and 6 months after surgery^11,16^. The Izbicki pain score is calculated via a validated questionnaire designed to assess pain in chronic pancreatitis and has been widely used in previous trials^5–7,11,17^. Secondary endpoints included pancreatic morphology, quality of life, postoperative events, perioperative and postoperative outcomes, and pain relief at the end of follow-up. Postoperative outcomes included length of hospital stay, minor and major complications (using the Clavien–Dindo classification)^18^, type of surgical technique, procedure volume, and death. Quality of life was assessed using Pancreatitis Quality of Life Instrument (PANQOLI) and 12-Item Short-Form (SF-12) surveys, the latter giving a Physical Component Summary (PCS) score and a Mental Component Summary (MCS) score^19,20^. All outcomes within the context of pain were only analysed for patients who had intractable pain or frequent pain flares as an indication for surgery.

### Definitions

Complications of surgical treatment were reported using the International Study Group for Pancreatic Surgery (ISGPS) definitions^21^. Only ISGPS grade B/C complications were included. Postoperative complications were also reported using the Clavien–Dindo classification of surgical complications^18^. Major complications were defined as grade IIIa or higher. Complications, readmissions, and deaths were all recorded up to 90 days after surgery. Data on pancreatic insufficiency (that is endocrine and exocrine) were collected before surgery. Endocrine insufficiency was defined by the use of antidiabetic medication and exocrine insufficiency was defined by the use of pancreatic enzyme replacement therapy. A toxic/metabolic aetiology of chronic pancreatitis included the use of alcohol, tobacco smoking, hypercalcaemia, hyperlipidaemia, medication, and toxins. Other types of pain medication comprised non-opioid pain medication and included paracetamol, non-steroidal anti-inflammatory drugs (NSAIDs), and neuropathic analgesics, such as gabapentin. Pain relief was assessed using the definitions previously used in the ESCAPE trial; complete pain relief was defined as an Izbicki pain score ≤10 and partial pain relief was defined as an Izbicki pain score >10, but with a >50% decrease compared with the baseline score^5^.

A pancreatic head with a diameter of >4 cm was considered enlarged^11^ and a main PD with a diameter of ≥5 mm was considered dilated^22^. Pancreatic morphology of chronic pancreatitis was described as ‘normal’ for a small PD and small pancreatic head, ‘solely dilated main PD’, ‘enlarged pancreatic head’, or ’other’ (for example pseudocysts and groove pancreatitis).

Aetiology was reported according to TIGAR-O classification^23^. Indications to perform surgery were reported as either pain (defined as intractable pain or frequent pain flares) or complications due to chronic pancreatitis (defined as common bile duct (CBD) obstruction, duodenal or bowel obstruction, the presence of pseudocysts, and other).

### Surgical techniques

**Fig. 1:**
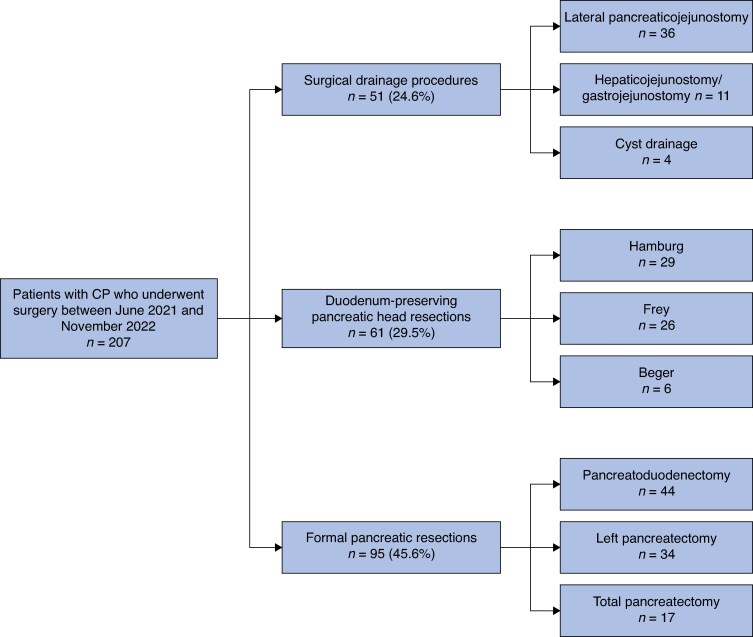
Overview of surgical procedures CP, chronic pancreatitis.

**Fig. 2:**
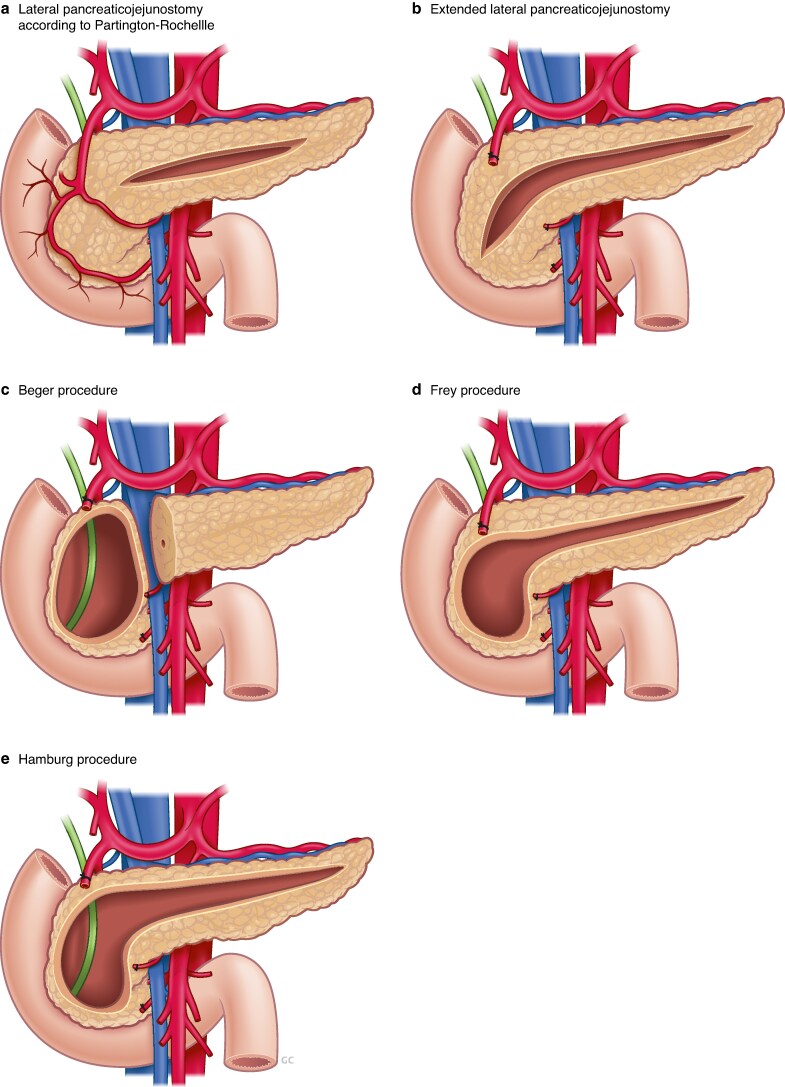
Surgical techniques for chronic pancreatitis **a** Lateral pancreaticojejunostomy according to Partington-Rochelle. **b** Extended lateral pancreaticojejunostomy. **c** Beger procedure. **d** Frey procedure. **e** Hamburg procedure. © van der Zon - Visueel.

Surgical treatment for chronic pancreatitis was classified into three categories: surgical drainage procedures, DPPHRs, and formal pancreatic resections. Surgical drainage procedures included lateral pancreaticojejunostomy (LPJ), which included extended LPJ^24^, as well as Partington-Rochelle, Puestow, hepaticojejunostomy (HJ), gastrojejunostomy (GJ), and pseudocyst drainage procedures. No distinction was made between extended LPJ and ‘standard’ LPJ for the purpose of the present study. DPPHRs included Frey, Beger, Bern, and Hamburg procedures^16,21,25,26^. Formal pancreatic resections included pancreatoduodenectomy, left pancreatectomy, and total pancreatectomy with or without islet autotransplantation. The *supplementary methods*, *Fig. 1*, and *Fig. 2* provide an overview of the different surgical techniques. The type of surgical procedure chosen for a particular patient was at the discretion of the attending surgeon.

### Data collection

Each participating centre assigned one person for data collection and local coordination. An online electronic case report form (eCRF) (Electronic Data Capture (EDC) System, Castor Amsterdam, The Netherlands) was used^10^; it captured information on baseline characteristics and the morphology of the pancreas, as well as intraoperative, postoperative, morbidity, and death information. Before inclusion, completion of a questionnaire on ‘local standard of care’ by the physician and completion of an informed consent form (ICF) by the patient were required. At baseline and subsequent visits (1, 3, and 6 months), a link to the questionnaires regarding pain and quality of life were sent using Castor’s EDC System. If patients had no access to online questionnaires, patients were followed up by telephone by the local surgeon or during outpatient clinical visits. An automatic reminder was sent through Castor’s EDC System if the questionnaire was not completed within 7 days and a maximum of two reminders were sent. Three weeks after the initial invitation, questionnaires were closed and reported as missing data if the questionnaires had not been completed.

### Statistical analyses

SPSS^®^ (IBM, Armonk, NY. USA; Statistics, version 28) was used to execute the statistical analyses. Preoperative and postoperative characteristics are presented using descriptive statistics and were assessed using one-way ANOVA, the Kruskal–Wallis test, the chi-squared test, Fisher’s exact test, or the Wilcoxon signed-rank test, as appropriate. Additional logistic regression analyses were performed to identify predictors of pain relief at 6 months. Based on previous literature the following variables were entered into the model: duration of symptoms, preoperative opioid use, prior endoscopic intervention, current tobacco and alcohol use, and type of surgery^27,28^. The results are reported as OR (95% c.i.). A linear mixed model was employed to analyse the mean Izbicki pain score during follow-up. The moment of follow-up (that is baseline or 1, 3, or 6 months after surgery) was included as a covariate and the annual volume of pancreatic surgery was included as a fixed effect to assess its impact on the Izbicki pain score over time. Different covariance structures were compared using the Akaike Information Criterion (AIC) to determine optimal model fit.

All *P* values are two-tailed and *P* < 0.050 was considered statistically significant.

## Results

### Baseline characteristics

Between 1 June 2021 and 30 November 2022, 232 patients were identified as scheduled to undergo surgery for symptomatic chronic pancreatitis. Of these, 25 patients were excluded for the following reasons: no surgical intervention within the enrolment interval (19 patients), pathologically proven pancreatic cancer (3 patients), missing baseline data (2 patients), and withdrawal of informed consent (1 patient). See *Fig. S2*. Finally, 207 patients were included from 22 centres in 13 European countries; 128 of these patients (61.8%) were male and the mean(s.d.) age was 50.0(13.9) years. A toxic/metabolic aetiology was the most frequently reported aetiology of chronic pancreatitis (86 patients (41.5%)), with intractable pain and frequent pain flares as the main indication for surgery (75.8%). In 148 patients (71.5%), the PD was dilated, whereas the pancreatic head was enlarged in 56 patients (27.7%). Alcohol use at time of surgery was reported in 27 patients (13.0%) and tobacco use at time of surgery was reported in 104 patients (50.2%).

The median time between the diagnosis of chronic pancreatitis and surgery was 11.0 (interquartile range (i.q.r.) 5.0–36.0) months. Endocrine insufficiency was present in 61 patients (29.5%) and exocrine insufficiency was present in 107 patients (51.7%). Opioids were used by 101 patients (48.8%) before surgery, with 30.0% using opioids for ≥12 months, and 106 patients (51.2%) had undergone prior endoscopic treatment (see *Table S1*). The median baseline Izbicki pain score was 61.3 (i.q.r. 49.1–84.9) and the mean(s.d.) quality-of-life PANQOLI, SF-12 PCS, and SF-12 MCS scores were 56.8(17.1), 37.2(12.0), and 39.3(7.2) respectively. Baseline characteristics are presented in *Table 1*.

**Table 1:** Baseline characteristics; total number of patients = 207

	Value
**Sex**	
Male	128 (61.8)
Female	79 (38.2)
Age at surgery (years), mean(s.d.)	50.0 (13.9)
BMI (kg/m^2^), mean(s.d.)	23.6 (4.1)
Duration of symptoms (months), median (i.q.r.)*	11.0 (5.0–36.0)
**Total Charlson co-morbidity index score†**	
0	71 (34.3)
1–2	86 (41.5)
3–4	34 (16.4)
≥5	14 (6.8)
**Aetiology**	
Toxic/metabolic‡	86 (41.5)
Idiopathic	32 (15.5)
Genetic	20 (9.7)
Autoimmune	6 (2.9)
Recurrent and severe acute pancreatitis	19 (9.2)
Obstructive	26 (12.6)
Other/unknown	18 (8.7)
**Indication for surgery**	
Intractable pain/frequent pain flares	157 (75.8)
Common bile duct obstruction	24 (11.6)
Pancreatic pseudocyst	41 (19.8)
Pancreatic ascites	8 (3.9)
Duodenal obstruction	20 (9.7)
Vascular complications	5 (2.4)
Pancreatic cancer cannot be fully excluded	28 (13.5)
Redo surgery for CP	8 (3.9)
Other	18 (8.7)
Dilated pancreatic duct (diameter ≥5 mm)	148 (71.5)
Enlarged pancreatic head (diameter >4 cm)§	56 (27.7)
‘Small duct pancreatitis’ (normal duct and head)	48 (23.2)
Current alcohol use¶	27 (13.0)
Current tobacco use¶	104 (50.2)
Endocrine insufficiency¶	61 (29.5)
Exocrine insufficiency¶	107 (51.7)
Use of opioids before surgery	101 (48.8)
**Duration of opioid use (months)#**	
<1	5 (5.0)
1–2	20 (19.8)
3–6	26 (25.7)
7–12	19 (18.8)
≥12	30 (29.7)
**Prior endoscopic intervention for CP**	106 (51.2)
Number of procedures, median (i.q.r.)	3.0 (2.0–4.0)
Izbicki pain score at baseline, median (i.q.r.)**	61.3 (49.1–84.9)
PANQOLI score at baseline, mean(s.d.)††	56.8 (17.1)
**SF-12 score at baseline, mean(s.d.)‡‡**	
Physical Component Summary score	37.2 (12.0)
Mental Component Summary score	39.3 (7.2)
**Annual volume of surgery for CP (number of procedures per year)§§**	
<10	12 (55)
10–14	3 (13)
≥15	6 (27)
**Annual volume of pancreatic surgery (number of procedures per year)§§**	
<49	8 (36)
50–99	9 (41)
≥100	4 (18)

Values are *n* (%) unless otherwise indicated. *Information missing for seven patients. †Information missing for two patients. ‡Including alcohol and tobacco use, medication, hypercalcaemia, hyperlipidaemia, and toxins. §Not applicable for five patients. ¶Use at time of surgery. #Information missing for one patient. **Only calculated for patients with pain as an indication for surgery and complete questionnaires at baseline and 6-month follow-up (113 patients). ††Only calculated for patients with complete questionnaires at baseline and 6-month follow-up (170 patients). ‡‡Only calculated for patients with complete questionnaires at baseline and 6-month follow-up (168 patients). §§Information missing for 1 of the 22 centres. i.q.r., interquartile range; CP, chronic pancreatitis; PANQOLI, Pancreatitis Quality of Life Instrument; SF-12, 12-Item Short-Form.

### Surgical practices

The annual volume of surgery for chronic pancreatitis was <10 procedures in 12 centres and ≥10 procedures in 9 centres; this information was missing for 1 of the 22 centres. The annual volume of pancreatoduodenectomy (for all indications) was <49 procedures in 8 centres (36%), 50–99 procedures in 9 centres (41%), and ≥100 procedures in 4 centres (18%); this information was missing for 1 of the 22 centres. In *Table S4*, characteristics of surgery for chronic pancreatitis in countries including ≥10 patients are compared.

Of the 207 patients undergoing surgery for chronic pancreatitis, 51 (24.6%) underwent surgical drainage procedures, 61 (29.5%) underwent DPPHRs, and 95 (45.9%) underwent formal pancreatic resections. In the surgical drainage group, most patients underwent an (extended) LPJ (36 of 51 (70.6%)). Within the DPPHR group, 29 patients underwent a Hamburg procedure, 26 patients underwent a Frey procedure, and 6 patients underwent a Beger procedure. Within the formal pancreatic resection group, 44 patients underwent pancreatoduodenectomy, 34 patients underwent left pancreatectomy, and 17 patients underwent total pancreatectomy. See *Fig. 1*. The type of surgical procedure used for chronic pancreatitis varied significantly per country (*P* < 0.001). An open surgical approach was used in most patients (182 patients (87.9%)).

### Surgical outcomes

Perioperative and postoperative outcomes are presented in *Table 2*. Major complications occurred in 29 patients (14.0%). The median hospital stay was 11.0 (i.q.r. 8.0–17.0) days. Within 90 days after index surgery, 30 patients (14.5%) were readmitted, 18 patients (8.7%) required surgical, endoscopic, or radiological re-intervention, and 3 patients (1.4%) died (all related to the initial pancreatic surgery). During follow-up, but after 90 days, one additional patient (0.5%) died, which was not related to the index procedure.

**Table 2:** Outcomes of surgery for symptomatic chronic pancreatitis (overall and stratified by type of surgery)

	Overall (*n* = 207)	Surgical drainage procedures (*n* = 51)	DPPHRs (*n* = 61)	Formal pancreatic resections (*n* = 95)	*P*
**Surgical approach**					
Open	182 (87.9)	46 (90)	56 (92)	80 (84)	0.309‡
Laparoscopic	19 (9.2)	4 (8)	4 (7)	11 (12)	0.551‡
Robot assisted	6 (2.9)	1 (2.0)	1 (2)	4 (4)	0.660‡
Use of opioids before surgery	101 (48.8)	24 (47)	37 (61)	40 (42)	0.074‡
Endocrine insufficiency at time of surgery	61 (29.5)	19 (37)	18 (30)	24 (25)	0.317‡
Exocrine insufficiency at time of surgery	107 (51.7)	23 (45)	39 (64)	45 (47)	0.072‡
Dilated pancreatic duct (diameter ≥5 mm)	148 (71.5)	43 (84)	48 (79)	57 (60)	0.003‡§
Enlarged pancreatic head (diameter >4 cm)*	56 (27.7)	11 (22)	24 (39)	21 (23)	0.053‡
‘Small duct pancreatitis’ (normal duct and head)	48 (23.2)	7 (14)	10 (16)	31 (33)	0.012‡§
**Ninety-day outcomes**					
Major complications (Clavien–Dindo grade ≥III)	29 (14.0)	4 (8)	11 (18)	14 (15)	0.287‡
ISGPS-specific complications (grade B/C)†					
Postoperative pancreatic fistula	23 (32.4)	3 (25)	9 (38)	11 (31)	0.837‡
Delayed gastric emptying	8 (11.3)	3 (25)	3 (13)	2 (6)	0.130‡
Chyle leak	3 (4.2)	0 (0.0)	1 (4)	2 (6)	>0.999‡
Post-pancreatectomy haemorrhage	5 (7.0)	0 (0.0)	3 (13)	2 (6)	0.467‡
Hospital stay (days), median (i.q.r.)	11.0 (8.0–17.0)	9.0 (7.0–12.0)	10.0 (7.0–15.0)	13.0 (9.0–20.0)	0.012¶§
Readmission	30 (14.5)	4 (8)	15 (25)	11 (12)	0.033‡§
Re-intervention	18 (8.7)	2 (4)	10 (16)	6 (6)	0.050‡§
Death	3 (1.4)	0 (0.0)	2 (3)	1 (1)	0.454‡

Values are *n* (%) unless otherwise indicated. All percentages reflect the total number of patients per subgroup, including patients with missing information. *Not applicable for five patients. †All definitions according to the ISGPS. ‡A chi-squared or Fisher’s exact test was used for categorical variables. §Statistically significant. ¶A Kruskall–Wallis test was used for data with a non-normal distribution. DPPHRs, duodenum-preserving pancreatic head resections; ISGPS, International Study Group for Pancreatic Surgery; i.q.r., interquartile range.

### Pain outcomes

The median Izbicki pain score improved from 61.3 (i.q.r. 49.1–84.9) at baseline to 19.0 (i.q.r. 0.0–33.5) at 6-month follow-up (*P* < 0.001). At 6-month follow-up, reduced pain scores were reported by 102 of 113 patients (90.3%) who were operated on for pain. Among these 113 patients, complete pain relief was reported by 43 patients (38.1%) and partial pain relief was reported by 39 patients (34.5%). Between the three surgical groups, the rates of complete pain relief (*P* = 0.308) and partial pain relief (*P* = 0.248) did not differ. Izbicki pain scores at baseline and at 6-month follow-up did not differ between the surgical groups (*P* = 0.205 and *P* = 0.428 respectively). See *Table 3* and *Table S3*.

**Table 3:** Functional outcomes at 6-month follow-up (overall and stratified by type of surgery)

	Overall (*n* = 207)	Surgical drainage procedures(*n* = 51)	DPPHRs (*n* = 61)	Formal pancreatic resections (*n* = 95)	*P*
**Primary outcomes**					
Izbicki pain score at 6-month follow-up, median (i.q.r.)*	19.0 (0.0–33.5)	19.0 (0.0–31.0)	15.0 (0.0–33.8)	21.0 (1.5–34.0)	0.428§
Pain relief at 6-month follow-up*	82 (72.6)	20 (74)	26 (68)	36 (75)	0.502¶
Complete	43 (38.1)	10 (37)	18 (47)	15 (31)	0.308¶
Partial	39 (34.5)	10 (59)	8 (40)	21 (64)	0.248¶
**Secondary outcomes**					
VAS score at 6-month follow-up, median (i.q.r.)*	2.0 (0.0–26.0)	10.0 (1.0–30.0)	1.5 (0.0–30.0)	1.0 (0.0–20.0)	0.099§
Change from baseline to 6-month follow-up					
VAS score, median (i.q.r.)*	53.5 (30.0–77.0)	50.0 (31.0–71.0)	52.0 (21.0–78.0)	60.0 (35.0–80.0)	0.253§
PANQOLI score, mean(s.d.)†	24.4 (22.3)	25.4 (25.1)	27.8 (21.6)	21.7 (21.3)	0.290#
SF-12 score, mean(s.d.)‡					
** ** Physical Component Summary score	14.4 (14.8)	14.9 (18.4)	16.4 (15.8)	13.0 (12.7)	0.442#
** ** Mental Component Summary score	−4.6 (9.5)	12.8 (15.3)	12.4 (12.4)	12.0 (12.7)	0.951#

Values are *n* (%) unless otherwise indicated. All percentages reflect the total number of patients per subgroup, including patients with missing information. *Only calculated for patients with pain as an indication for surgery and with complete questionnaires at baseline and 6-month follow-up (113 patients). †Only calculated for patients with complete questionnaires at baseline and 6-month follow-up (170 patients). ‡Only calculated for patients with complete questionnaires at baseline and 6-month follow-up (168 patients). §A Kruskall–Wallis test was used for data with a non-normal distribution. ¶A chi-squared test or Fisher’s exact test was used for categorical variables. #One-way ANOVA was used for data with a normal distribution. DPPHRs, duodenum-preserving pancreatic head resections; i.q.r., interquartile range; VAS, Visual Analogue Scale; PANQOLI, Pancreatitis Quality of Life Instrument; SF-12, 12-Item Short-Form.

The median decrease in Visual Analogue Scale score (0–100) was 53.5 (i.q.r. 30.0–77.0) and did not differ among the groups: surgical drainage procedures, 50.0 (i.q.r. 31.0–71.0); DPPHRs, 52.0 (i.q.r. 21.0–78.0); and formal pancreatic resections, 60.0 (i.q.r. 35.0–80.0) (*P* = 0.253).

### Predictors of complete pain relief

**Fig. 3:**
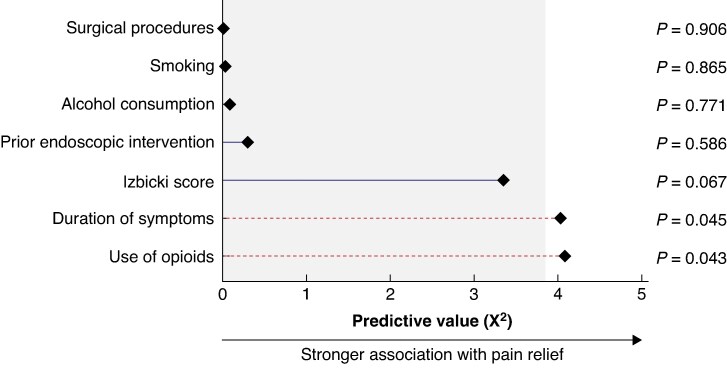
Predictors for complete pain relief

A multivariable analysis showed that longer duration of symptoms (OR 0.95 (95% c.i. 0.90 to 1.00), *P* = 0.045) and use of opioids before surgery (OR 3.16 (95% c.i. 1.04 to 9.64), *P* = 0.043) were significantly associated with less pain relief after 6 months. None of the other predictors, including Izbicki pain score at baseline, prior endoscopic intervention, type of surgical procedure, and tobacco and alcohol use at time of surgery, was significantly associated with pain relief. See *Table S2* and *Fig. 3*. In addition, duration of symptoms exceeding the 75th percentile (41 months) was associated with a lower likelihood of complete pain relief (*P* = 0.037). In contrast, preoperative opioid use of >6 months did not show a similar association (*P* = 0.302). See *Table S6*. Linear mixed model analyses demonstrated a significant association between duration of follow-up and decrease in Izbicki pain score (*P* < 0.001). See *Fig. S1*.

### Quality of life

For both PANQOLI and SF-12 scores, no differences between the different surgical groups were seen at baseline and at 6-month follow-up. The mean(s.d.) PANQOLI score improved significantly from 56.8(17.1) to 81.1(15.9) after 6 months (*P* < 0.001). Within SF-12, the mean(s.d.) PCS score improved from 37.2(12.0) to 51.6(10.7) (*P* < 0.001), whereas the mean(s.d.) MCS score decreased significantly from 39.3(7.2) to 34.7(7.4) (*P* < 0.001). See *Table 3* and *Table S3*. In patients without pain, similar trends were observed for functional outcomes. Overall, the median PANQOLI score improved from 67.0 (i.q.r. 53.0–81.0) to 87.0 (i.q.r. 77.0–93.0) (*P* < 0.001) and, within SF-12, the median PCS score improved from 43.4 (i.q.r. 35.2–52.9) to 56.5 (i.q.r. 47.3–60.9) (*P* < 0.001), whereas the median MCS score decreased from 37.0 (i.q.r. 32.8–41.0) to 33.6 (i.q.r. 28.9–39.5) (*P* = 0.024). See *Table S5*.

### Comparing surgical procedures

Surgical drainage procedures were associated with significantly lower rates of 90-day readmissions (surgical drainage procedures, 8%; DPPHRs, 25%; and formal pancreatic resections, 12% (*P* = 0.033)) and re-interventions (surgical drainage procedures, 4%; DPPHRs, 16%; and formal pancreatic resections, 6% (*P* = 0.050)), and a non-significant lower rate of mortality (surgical drainage procedures, 0%; DPPHRs, 3%; and formal pancreatic resections, 1% (*P* = 0.454)).

The absolute largest risk reduction for LPJ was 10.2% for major complications and 3.3% for death, indicating that, by treating approximately 30 eligible patients (with a dilated PD and a pancreatic head of normal size) with LPJ compared with DPPHR or formal pancreatic resection, major complications may be avoided in approximately 3 patients and death may be avoided in approximately 1 patient. See *Table S7*.

## Discussion

This prospective pan-European study confirms that surgery for symptomatic chronic pancreatitis is safe and effective, with pain relief after 6 months in most patients, as >90% reported reduced pain scores. Predictors for complete pain relief include a shorter duration of symptoms and the absence of opioid use before surgery, highlighting the importance of multidisciplinary consultation before any intervention in patients with symptomatic chronic pancreatitis.

Rates of major morbidity (14.0%) and 90-day mortality (1.4%) were low and largely comparable among the surgical groups. The safety profile was best for the surgical drainage group (51 patients), including a 90-day mortality rate of 0.0%. Izbicki pain scores improved significantly from a median of 61.3 to 19.0 after 6 months, with pain relief in 72.6% of patients operated on for pain and reduced pain scores reported by 90.3% of patients operated on for pain. Quality of life improved regarding physical symptoms and did not differ significantly among the three surgical groups. Longer duration of symptoms and use of opioids before surgery were associated with less pain relief after 6 months.

The present results can only be compared with retrospective studies and individual randomized trials, as international prospective multicentre studies have not been performed. Previous retrospective series also reported low rates of major morbidity (<20%) and mortality (<4%) after surgery for symptomatic chronic pancreatitis^27,29–31^. The present study confirms that drainage surgery (including (extended) LPJ) has the best safety profile. The same was reported by a recent Dutch nationwide retrospective study^28^. Current guidelines also advise to tailor surgery to chronic pancreatitis morphology and use the least invasive procedure when possible (that is drainage rather than resection)^32^. This stands out even more given that pain outcomes were comparable across surgical groups, whereas surgical morbidity was not.

The primary endpoint, the Izbicki pain score, varied widely at baseline measurement from 42.5 to 85.3 between individual countries, as did the proportion of patients using opioids before surgery (6.7% to 86.7%). This suggests that different thresholds are used for surgical intervention between European countries, which should be addressed by future studies. In addition, the pancreatic morphology (that is an enlarged pancreatic head and a dilated PD) of patients undergoing surgery for chronic pancreatitis differed among countries. A previous bi-national study that compared pancreatic morphology among patients undergoing surgery for chronic pancreatitis in Germany and the USA found more enlarged heads in German patients^33^. However, in the present study, the number of included patients per country varied from 12 to 77, and local pain protocols were not documented, and therefore this finding should be interpreted with some caution.

In line with previous studies^11,28^, good to excellent clinical outcomes were observed in terms of pain relief and quality of life in the present study. At 6-month follow-up, 72.6% of patients reported pain relief (38.1% of patients reported complete pain relief and 34.5% of patients partial pain relief). The multivariable analysis found comparable association with previously identified predictors for less pain relief (that is longer duration of symptoms and use of opioids before surgery; *Table S2*). This seems consistent with the theory of pain sensitization, where prolonged inadequate management of pain in chronic pancreatitis may cause pain to become less responsive to (surgical) interventions due to neuroplastic changes^34,35^. Further analysis demonstrated that a symptom duration exceeding the 75th percentile (>41 months) was associated with a lower likelihood of achieving complete pain relief (19.2% *versus* 43.7%, *P* = 0.037). These findings underline the importance of early referral for surgical treatment in patients with symptomatic chronic pancreatitis to optimize outcomes, rather than waiting until severe pain requiring opioids has developed, although this needs to be confirmed in future studies. In addition, among the 11 patients who reported a worsened Izbicki pain score 6 months after surgery, 9 (82%) had used opioids before surgery and 8 (72%) underwent prior endoscopic intervention.

Quality of life improved in terms of the PANQOLI score and the physical aspect of SF-12 (that is the PCS score); however, a decreased SF-12 MCS score was seen at the end of follow-up. Based on this, surgical treatment seems mostly effective in reducing physical symptoms and more attention should be given to the mental aspects of patients’ well-being during follow-up after surgery. Quality of life in general is a concern in these patients, which is reflected at both baseline and end of follow-up, with worse scores compared with the general population; a PCS score <50 and an MCS score <42 are seen as cut-offs^20^.

The present study shows that surgery for chronic pancreatitis remains relatively rare, with 207 procedures performed across 22 centres over an 18-month interval. The COVID-19 pandemic likely contributed to this low number, as benign pancreatic surgery was often postponed. Nonetheless, survey data indicate that most centres perform <10 surgical procedures for chronic pancreatitis annually. Similar findings were seen in a recent multicentre cross-sectional study from the Scandinavian Baltic Pancreatic Club, where only 95 of 1327 patients with chronic pancreatitis (7%) underwent surgery between 2016 and 2019^36^. Notably, the total number of procedures performed for chronic pancreatitis varied widely per centre in the present study (1–77), which resembles large differences in volume seen for oncological pancreatic resections across Europe.

The use of endoscopic treatment before surgery was assessed, revealing that half of the patients had undergone endoscopic treatment before surgery, with a median of 3 (i.q.r. 2–4) procedures per patient (*Table S1*). This finding was somewhat unexpected, as multiple studies have demonstrated the superiority of early surgical intervention over an endoscopy-first approach^5,37^. Nonetheless, the present findings align with a recent nationwide study from the DPSG^28^. Further research into this trend is currently being conducted through the ESCOPA-Endoscopy study, which is evaluating the use and outcomes of endoscopic treatment for chronic pancreatitis across Europe.

Another interesting finding is that 12.1% of procedures were performed using minimally invasive surgery (9.2% of procedures were laparoscopic and 2.9% of procedures were robot assisted). The benefits of minimally invasive surgery include less postoperative pain and faster time to recovery, and could therefore also be valuable in this category of patients with desensitization to pain medication, but randomized studies are lacking^38,39^. Taken together, the good results reported here may affect both surgeons’ and gastroenterologists’ preferences and could have implications for future surgical decision-making in patients with symptomatic chronic pancreatitis, leading to increased use of surgical procedures^40,41^.

The results of this study should be interpreted considering several limitations. First, participation in this study was voluntary and based on invitation to E-AHPBA members. This may have introduced bias, as less experienced centres could have been less likely to participate. However, the participating centres seemed balanced across low-, medium-, and high-volume centres. Second, four measurement points for pain evaluation were used. Interpretation of these results should be done with some caution, as pain patterns in chronic pancreatitis differ widely^42,43^. This led to a relatively shorter follow-up interval of 6 months after surgery. Consequently, long-term follow-up is needed. Interestingly, the recent 8-year follow-up of the ESCAPE trial clearly confirmed the long-term efficacy of surgery and its superiority over endoscopy^37^. Third, pain was assessed solely using the Izbicki pain score, which limits the evaluation of other pain domains. Future studies should also include other pain assessment tools to allow for a more complete evaluation. Fourth, 41 patients were lost to follow-up regarding the primary endpoint. The baseline pain scores of these 41 patients did not differ from the other patients (median Izbicki pain score of 65.8 (i.q.r. 43.5–78.8) *versus* 61.3 (i.q.r. 49.1–84.9), *P* = 0.959). Fifth, comparison of data in terms of indications, outcomes, and complications was difficult, as the design of the study permitted all indications for surgery. In addition, each hospital had slightly different treatment preferences. Future studies should therefore collect data on the surgical decision-making process, including standardized local pain protocols. Sixth, for evaluation of pancreatic morphology on preoperative imaging, the authors did not involve an independent radiologist to re-evaluate the imaging. The main strength of this study is that it represents the first international prospective study on surgery for chronic pancreatitis in a relatively large cohort of patients with symptomatic chronic pancreatitis.

## Supplementary Material

znaf068_Supplementary_Data

## Data Availability

Requests for data can be made to the corresponding author and will be discussed during a meeting of the Dutch Pancreatitis Study Group. After approval by the Dutch Pancreatitis Study Group, data that underlie the results reported in this study will be shared.

## Supplementary material

*Supplementary*  *material* is available at *BJS* online.
