# Supplementary material for: Surgery for chronic pancreatitis across Europe (ESCOPA): prospective multicentre study
Source: Br J Surg. 2025 Apr 29;112(4):znaf068. doi: 10.1093/bjs/znaf068 (PMC12038157; doi:10.1093/bjs/znaf068)

**Surgery for chronic pancreatitis across Europe (ESCOPA): prospective international multicentre study**

Charlotte L. van Veldhuisen ^1,2,3, #^, Charlotte A. Leseman^1,2,#^, Fleur E.M. De Rijk^2,4^, Joana C. Antunes^5^, Fabio Ausania^6^, Orlin Belyaev^7^, Frederik Berrevoet^8^, Marja A. Boermeester^1,2^, Ugo Boggi^9^, Stefan A. Bouwense^10^, Marco J. Bruno^4^ , Olivier R. Busch^1,2^, Kevin C. Conlon^11^, Safi Dokmak^12^, Massimo Falconi^13^, Poya Ghorbani^14^, Filip Gryspeerdt^8^, Roel Haen^15^, Arturan Ibrahimli^16^,Jakob R. Izbicki^17^, Christina Krikke^18^, Arto Kokkola^19^, Lancelot Marique^12,^, J. Sven D. Mieog^20^, Gennaro Nappo^21,22^, Janis Pavulans^23^, Haralds Plaudis^23^, Geert Roeyen^24^, Pasquale Scognamiglio^17^, Domenico Tamburrino^13^, Tore Tholfsen^25^, Marie Toschka^7^, Faik G. Uzunoglu^17^, Susan van Dieren^1,2^, Casper H.J. Van Eijck^15^, Jeanin E. van Hooft^26^, Hjalmar C. van Santvoort^27^, Robert C. Verdonk^28^, Rogier P. Voermans^29^, Anne Waage^25^, Marc G. Besselink^1,2^ for the Dutch Pancreatitis Study Group (DSPG) and the Scientific and Research Committee of the European-African Hepato-Pancreato-Biliary Association

(E-AHPBA)

^#^shared first authorship

^1^ Amsterdam UMC, location University of Amsterdam, Department of Surgery, Amsterdam, the Netherlands.

^2^ Amsterdam Gastroenterology Endocrinology Metabolism, the Netherlands.

^3^ Department of Research and Development, St. Antonius Hospital, Nieuwegein, the Netherlands.
^4^ Department of Gastroenterology and Hepatology, Erasmus University Medical Center, Rotterdam, the Netherlands.

^5^ Institution of Investigation, University of Porto, Rua Alfredo Allen, Porto, Portugal.

^6^ Department of Hepatic, Pancreatic, Biliary and Transplant Surgery, Clinic Hospital, University of Barcelona, IDIBAPS, Barcelona, Spain.

^7^ Department of Surgery, St. Josef-Hospital, University, Ruhr-Universität Bochum, Bochum, Germany.

^8^ General and HPB Surgery and Liver Transplantations, Ghent University Hospital, C. Heymanslaan 10, 9000, Ghent, Belgium.

^9^ Division of General and Transplant Surgery, University of Pisa, Pisa, Italy.

^10^ Department of Surgery, Maastricht University Medical Center+, Maastricht, the Netherlands.

^11^ Department of Surgery, Trinity College Dublin, Tallaght Hospital, Dublin, Ireland.

^12^ Department of HPB Surgery and Liver Transplantation, Hôpital Beaujon, APHP; Université de Paris Cité. Centre de Recherche sur l'Inflammation, INSERM Unité Mixte de Recherche 1149, Clichy, France.

^13^ Pancreas Translational and Clinical Research Center, IRCCS San Raffaele Scientific Institute, Università Vita-Salute, Milan, Italy.

^14^ Department of Upper Abdominal Diseases, Karolinska University Hospital, Stockholm, Sweden.

^15^ Department of Surgery, Erasmus MC Cancer Institute, Rotterdam, the Netherlands.

^16^ Liv Bona Dea Hospital, Baku, Azerbaijan

^17^ Department for general, visceral and thoracic surgery, University Hospital Hamburg Eppendorf, Hamburg, Germany.

^18^ Department of Surgery, University Medical Center Groningen, University of Groningen, Groningen, the Netherlands.

^19^ Department of Surgery, Helsinki University Hospital and University of Helsinki, Helsinki, Finland.

^20^ Department of Surgery, Leiden University Medical Center, Leiden, the Netherlands.

^21^ Humanitas University, Department of Biomedical Sciences, Milan, Italy.

^22^ Pancreatic Surgery Unit, Humanitas Clinical and Research Center, IRCCS, Milan, Italy.

^23^ Department of Surgery, Riga East Clinical University Hospital, Riga, Latvia.

^24^ Department of Hepatobiliary Transplantation and Endocrine Surgery, Antwerp University Hospital and University of Antwerp, Edegem, Belgium.

^25^ Department of Hepato-Pancreato-Biliary Surgery, Oslo University Hospital, Rikshospitalet, Oslo, Norway.

^26^ Department of Gastroenterology and Hepatology, Leiden University Medical Center, Leiden, the Netherlands.

^27^ Department of Surgery, St Antonius Hospital, Nieuwegein, the Netherlands; Department of Surgery, University Medical Center Utrecht, Utrecht, the Netherlands.

^28^ Department of Gastroenterology and Hepatology, St Antonius Hospital, Nieuwegein, the Netherlands.

^29^ Department of Gastroenterology and Hepatology, Amsterdam Gastroenterology Endocrinology Metabolism, Amsterdam UMC, University of Amsterdam, Amsterdam, the Netherlands.

**Corresponding author**

M.G. Besselink, MD MSc PhD

Amsterdam UMC, location University of Amsterdam

Department of Surgery, Cancer Center Amsterdam

De Boelelaan 1117 (ZH-7F), 1081 HV, The Netherlands

Telephone: +31-204444400

Mail: m.g.besselink@amsterdamUMC.nl

ORCID ID: https://orcid.org/0000-0003-2650-9350

**Supplementary Materials - Index**

| **Supplementary Methods** | |  | |  |
| --- | --- | --- | --- | --- |
| Supplement 1: Overview of surgical procedures | | *page 4-6* | |  |
| **Supplementary Figures and Tables** | |  | |  |
| Supplementary Table 1 Endoscopic procedures per surgical procedure  Supplementary Table 2 Multivariable analysis on predictors for complete pain relief  Supplementary Figure S1 Effect of surgery for symptomatic chronic pancreatitis on Izbicki pain score over time  Supplementary Table 3 Patient reported outcomes  Supplementary Table 4 Outcomes per country  Supplementary Table 5 Functional outcomes of patients without pain, total and stratified per type of surgery  Supplementary Table 6 Duration of symptoms and preoperative opioid use on pain relief  Supplementary Table 7 Surgery in patients with solely main pancreatic duct dilation | | *page 5*  *page 6*  *page 7*  *page 8*  *page 9*  *page 10*  *page 11* |  |  |
| Supplementary Figure S2 Flowchart of enrollment of study patients | | | *page 15* | |
|  |  | | |  |

**Supplementary Methods**

**Supplement 1: Overview of surgical procedures**

Below is a description of the surgical procedures performed in the present study. See Figure 2 for the most common techniques.

Drainage procedures

Drainage procedures aim at decompression of ductal hypertension, which are often indicated in patients with an enlarged pancreatic duct and normal size pancreatic head.

*Lateral pancreaticojejunostomy*

In the lateral pancreaticojejunostomy (LPJ) according to Partington-Rochelle (also known as the modified Puestow procedure) the pancreatic duct is opened over its entire length to the left of the gastroduodenal artery, without resection of pancreatic tissue. Reconstruction is performed by a single side-to-side anastomosis with a Roux-en-Y loop on the opened pancreatic duct.

*Extended lateral pancreaticojejunostomy*

In the extended LPJ, the entire main pancreatic duct is opened over nearly the entire length which includes suture closing of the gastroduodenal arterial arcade aiming to minimize the risk of disease recurrence (Figure A). On both sides of the pancreatic duct (superior and inferior border), the gastroduodenal artery is identified and ligated. The pancreatic duct is opened from approximately 10 mm before the papilla of Vater to 10-20mm before the tip of the pancreas. Reconstruction is similar to the conventional LPJ, whereby the full length of the opened pancreatic duct is overlaid with a proximal Roux-en-Y loop.

Formal pancreatic resection procedures

Resection procedures for chronic pancreatitis (CP) are predominantly indicated in patients with an inflammatory mass in the pancreatic head or in patients with complications as a result of CP (such as pseudocysts).

*Pancreatoduodenectomy*

Pancreatoduodenectomy for CP is usually performed in patients with groove pancreatitis or morphological abnormalities in the pancreatic head. See Figure D.

*Left pancreatectomy*

Left pancreatectomy for CP is usually performed in patients in whom morphological changes are limited to the pancreatic body and tail or only the pancreatic tail.

*Total pancreatectomy*

Total pancreatectomy for CP is often seen as last resort treatment option, for example in patients with extensive fibrosis without endoscopic treatment options. During total pancreatectomy, the entire pancreas and gallbladder are removed, and in some centers, is followed by reimplantation of the patients’ own islet cells in the portal circulation to preserve some part of the islet cell function. Total pancreatectomy and auto islet transplantation (TPIAT) was performed in 6 patients in the current study.

Duodenum preserving pancreatic head resections

DPPHR procedures combine both drainage and resection techniques, which are often effective in patients with enlargement of the pancreatic head and dilation of the pancreatic duct.

*Beger*

During the Beger procedure, the pancreatic neck is resected above the portal vein, followed by a subtotal resection of the diseased parenchyma in the pancreatic head. Subsequently, a rim of pancreatic tissue remains along the duodenum. Two end-to-end pancreaticojejunostomies complete the reconstruction: one on the pancreas remnant and one on the rim of pancreatic tissue along the duodenum.

*Frey*

Instead of a transection at the pancreatic neck, the pancreatic head is cored out and the pancreatic neck is preserved, leaving a rim of pancreatic tissue along the duodenum. The gastroduodenal artery is identified and ligated below and above the pancreatic duct. Next, the main pancreatic duct is drained via a longitudinal incision over the entire length. Reconstruction is accomplished by a single side-to-side pancreaticojejunostomy similar to an extended lateral pancreaticojejunostomy.

*Hamburg procedure*

The Hamburg procedure was described in detail by Izbicki et al.[1] In summary, to identify the intrapancreatic course of the distal common bile duct, a metal probe is placed into the common bile duct through a proximal choledochotomy. Starting from the upper and lower edges of the gland, the ventral pancreatic aspect is longitudinally excised, with the tip of the excised wedge being located deep in the dorsal part of the pancreas. The resulting longitudinal triangular cavity drains secondary and tertiary ductal branches into a Roux-en-Y loop, which is anastomosed to the pancreas as a pancreatojejunostomy using a single-layer monofilament running suture. In distal common bile duct stenosis, the intrapancreatic course of the choledochal duct is freed from fibrotic tissue.

**Supplementary Figures and Tables**

**Supplementary Table 1 Endoscopic procedures prior to chronic pancreatitis surgery**

|  | **Overall** (n=207) | **Surgical drainage procedures** (n=51) | **DPPHRs**  (n=61) | **Formal pancreatic resection** (n=95) | ***P*** |
| --- | --- | --- | --- | --- | --- |
| **Prior endoscopic procedure,** no. of patients (%) | 106 (51.2) | 30 (58.8) | 35 (57.4) | 41 (43.2) | 0.101^a^ |
| **Number of endoscopic procedures,** median (IQR) | 3 (2.0-4.0) | 2.0 (2.0-4.3) | 3.0 (2.0-4.0) | 3.0 (1.0-5.0) | 0.926^$^ |
| **Sphincterotomy**, no. (%) | 59 (28.5) | 17 (33.3) | 24 (39.3) | 18 (18.9) | 0.015^a^ |
| **ESWL**, no. (%) | 10 (4.8) | 4 (7.8) | 3 (4.9) | 3 (3.2) | 0.449^a^ |
| **Stent placement for PD strictures,** no. (%) | 54 (26.1) | 14 (27.5) | 16 (26.2) | 24 (25.3) | 0.959^a^ |
| **Stent placement for biliary strictures**, no. (%) | 20 (9.7) | 3 (5.9) | 6 (9.8) | 11 (11.6) | 0.599^a^ |
| **Drainage of pseudocysts,** no. (%) | 20 (9.7) | 6 (11.8) | 8 (13.1) | 6 (6.3) | 0.321^a^ |
| **Removal/exchange stents,** no. (%) | 30 (14.5) | 11 (21.6) | 7 (11.5) | 12 (12.6) | 0.250^a^ |
| **Other procedures**, no. (%)^±^ | 7 (3.4) | 4 (7.8) | 2 (3.3) | 1 (1.1) | 0.074^a^ |
| ^±^ Other procedures included: unsuccessful attempt ± papillotomy (3), cannulation (1), endoscopic EHL (1), insertion of jejunal feeding tube (1), transgastric drainage (1). ESWL = extracorporeal shockwave lithotripsy. ^a^ Chi-square or Fishers exact test was used for categorical variables.^$^ Kruskall-Wallis test was used for non-normal distributed data. All percentages are reflecting the total number of patients per subgroup including missing cases. | | | | | |

**Supplementary Table 2 Multivariable analysis on predictors for complete pain relief**

| **Predictors** | **OR [95% CI]** | ***p*-value** |
| --- | --- | --- |
| Izbicki score at baseline (per 10 points) | 1.231 [0.990-1.534] | 0.067 |
| Duration of symptoms in months (per 6 months) | 0.947 [0.897-1.000] | 0.045 |
| No use of opioids | 3.161 [1.036-9.643] | 0.043 |
| Prior endoscopic intervention | 1.296 [0.510-3.295] | 0.586 |
| Surgical procedure^#^ | 1.035 [0.584-1.836] | 0.906 |
| Smoking at time of surgery | 1.056 [0.561-1.989] | 0.865 |
| Drinking at time of surgery | 0.893 [0.418-1.911] | 0.771 |
| *^#^ Reference category is drainage surgery*  *Included in analysis: only patients with pain as indication for surgery and complete questionnaires at baseline and 6 months follow-up (n=109, missing cases n=4)* | *-2 Log likelihood 117.502, df = 7*  *Nagelkerke R Square 0.136* | |

**Supplementary Figure S1 Effect of surgery for symptomatic chronic pancreatitis on Izbicki pain score over time**


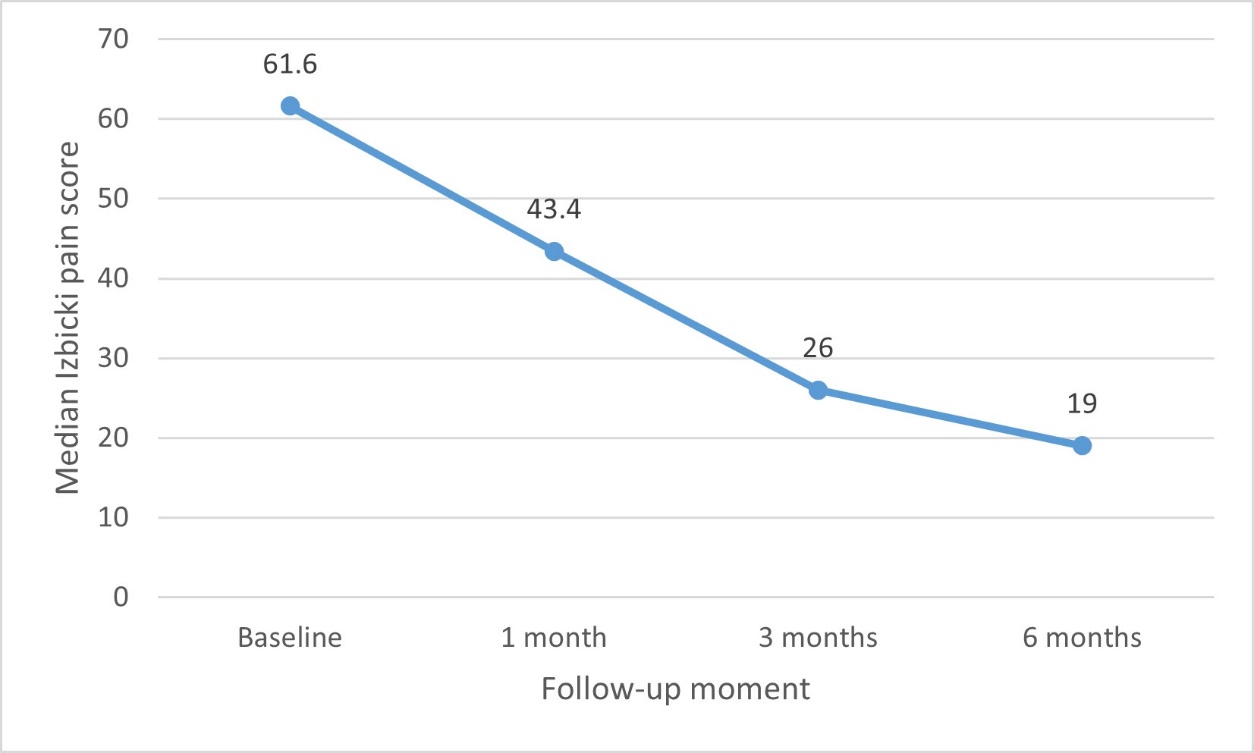


An additional linear mixed model confirmed that the moment of follow-up was significantly associated with Izbicki pain score (p <0.001).

**Supplementary Table 3** **Patient reported outcomes, overall and stratified per type of surgery**

|  | **Overall** (n=207) | **Surgical drainage procedures** (n=51) | **DPPHRs**  (n=61) | **Formal pancreatic resection** (n=95) | ***P*** |
| --- | --- | --- | --- | --- | --- |
| **Izbicki pain score at baseline**, median (IQR)^≠^ | 61.3 (49.1-84.9) | 68.8 (50.8-80.8) | 55.8 (47.1-70.0) | 63.6 (50.1-87.9) | 0.205^$^ |
| **VAS score at baseline**, median (IQR)^≠^ | 70.0 (50.0-80.0) | 70.0 (50.0-80.0) | 70.0 (40.0-80.0) | 72.5 (50.0-90.0) | 0.313^$^ |
| **PANQOLI score at baseline,** mean (SD)^¥^ | 56.8 (17.1) | 57.3 (19.4) | 54.4 (16.3) | 58.1 (16.7) | 0.479^β^ |
| Physical functioning | 18.3 (6.9) | 18.9 (6.8) | 16.6 (7.0) | 19.2 (6.8) | 0.088^β^ |
| Role function | 12.1 (3.9) | 11.8 (4.9) | 12.1 (3.4) | 12.4 (3.7) | 0.766^β^ |
| Emotional function | 12.0 (5.5) | 12.3 (6.0) | 11.8 (5.7) | 11.8 (5.3) | 0.901^β^ |
| Self-worth | 14.4 (4.9) | 14.3 (5.8) | 13.9 (4.1) | 14.7 (4.9) | 0.670^β^ |
| **PANQOLI score at 6 months**, mean (SD)^¥^ | 81.1 (15.9) | 82.6 (16.9) | 82.3 (17.0) | 79.8 (14.6) | 0.563^β^ |
| Physical functioning | 25.4 (4.9) | 25.5 (5.2) | 25.2 (5.4) | 25.5 (4.5) | 0.940^β^ |
| Role function | 18.0 (4.8) | 18.6 (5.1) | 18.7 (4.5) | 17.5 (4.8) | 0.282^β^ |
| Emotional function | 18.5 (5.4) | 19.2 (5.8) | 18.8 (5.4) | 18.0 (5.3) | 0.513^β^ |
| Self-worth | 19.3 (4.3) | 19.8 (4.5) | 19.7 (4.3) | 18.8 (4.2) | 0.361^β^ |
| **SF-12 score at baseline,** mean (SD)^µ^ |  |  |  |  |  |
| Physical Component Summary Score | 37.2 (12.0) | 35.8 (13.8) | 36.2 (12.2) | 38.3 (11.0) | 0.458^β^ |
| Mental Health Component Summary Score | 39.3 (7.2) | 37.9 (8.2) | 40.1 (7.4) | 39.4 (6.6) | 0.376^β^ |
| **SF-12 score at 6 months,** mean (SD)^µ^ |  |  |  |  |  |
| Physical Component Summary Score | 51.6 (10.7) | 50.7 (12.5) | 52.5 (10.6) | 51.3 (9.9) | 0.709^β^ |
| Mental Health Component Summary Score | 34.7 (7.4) | 35.0 (9.1) | 33.9 (6.1) | 35.0 (7.4) | 0.695^β^ |
| ^≠^ Only calculated for patients with pain as indication for surgery and with complete questionnaires at baseline and 6 months follow-up (n=113). ^¥^ Only calculated for patients with complete questionnaires at baseline and 6 months follow-up (n=170).  ^µ^  Only calculated for patients with complete questionnaires at baseline and 6 months follow-up (n=168) ^β^ One-way ANOVA was used for normal distributed data. ^$^ Kruskall-Wallis test was used for non-normal distributed data. All percentages are reflecting the total number of patients per subgroup including missing cases. | | | | | |

**Supplementary Table 4 Comparing countries with at least 10 patients included**

| **Country** | **No. patients** | **No. of patients using opioids**  **(%)** | **Most reported duration of opioid use**  **(%)** | **Most frequent indication for surgery**  **(%)** | **No. of patients with endoscopic intervention**  **(%)** | **Enlarged pancreatic head (>4cm)**  **(%)** | **Dilated main pancreatic duct (>5mm)**  **(%)** | **Surgical drainage procedures**  **(%)** | **DPPHRs**  **(%)** | **Formal pancreatic resection**  **(%)** | **Izbicki score at baseline***  **(IQR)** | **Complete pain relief* (%)** | **Partial pain relief***  **(%)** |
| --- | --- | --- | --- | --- | --- | --- | --- | --- | --- | --- | --- | --- | --- |
| Belgium | 15 | 6 (40.0) | < 6 months (60.0) | Pain (80.0) | 15 (100.0) | 2 (13.3) | 10 (66.7) | 7 (46.7) | 3 (20.0) | 5 (33.3) | 52.3 (38.1-82.3) | 6 (50.0) | 2 (16.7) |
| France | 12 | 8 (66.7) | > 12 months  (62.5) | Pain (66.7) | 4 (33.3) | 1 (8.3) | 9 (75.0) | 5 (41.7) | 4 (33.3) | 3 (25.0) | 66.3 (54.3-91.1) | 0 | 2 (25.0) |
| Germany | 77 | 25 (32.5) | < 6 months (28.0) | Pain (67.5) | 40 (51.9) | 13 (16.9) | 47 (61.0) | 10 (13.0) | 30 (39.0) | 37 (48.1) | 52.6 (39.1-74.1) | 15 (28.8) | 5 (9.6) |
| Italy | 15 | 1 (6.7) | < 6 months (100.0) | PDAC cannot be ruled out (33.3) | 5 (33.3) | 6 (54.4) | 11 (73.3) | 1 (6.7) | 0 | 14 (93.3) | 42.5 (23.6-60.8) | 3 (75.0) | 1 (25.0) |
| Latvia | 15 | 13 (86.7) | < 3 months (53.8) | Pseudocysts (40.0)  Duodenal obstruction (40.0) | 0 | 6 (40.0) | 14 (93.3) | 9 (46.9) | 5 (33.3) | 1 (6.7) | 71.3 (54.4-73.8) | 7 (46.7) | 5 (33.3) |
| The Netherlands | 31 | 25 (80.6) | > 12 months  (48.0) | Pain (93.5) | 21 (67.7) | 19 (61.3) | 26 (83.9) | 5 (16.1) | 15 (48.4) | 11 (35.5) | 70.0 (51.9-86.0) | 5 (17.9) | 11 (39.1) |
| Norway | 25 | 15 (60.0) | > 12 months  (46.7) | Pain (92.0) | 12 (48.0) | 1 (4.0) | 17 (68.0) | 8 (32.0) | 0 | 17 (68.0) | 85.3 (55.3-92.0) | 5 (16.7) | 9 (30.0) |

**Supplementary Table 5 Functional outcomes of patients without pain, overall and stratified per type of surgery**

|  | **Overall**  (n=207) | **Surgical drainage procedures** (n=51) | **DPPHRs**  (n=61) | **Formal pancreatic resection** (n=95) | ***P*** |
| --- | --- | --- | --- | --- | --- |
| **PANQOLI score at baseline,** median (IQR)^¥^ | 67.0 (53.0-81.0) | 80.0 (57.0-89.0) | 46.0 (41.3-75.5) | 65.0 (53.5-79.5) | 0.099^$^ |
| **PANQOLI score at 6 months**, median (IQR)^¥^ | 87.0 (77.0-93.0) | 93.0 (80.0-100.0) | 87.5 (79.3-97.3) | 86.0 (76.3-92.8) | 0.458^$^ |
| **Δ baseline – 6 months** | 14.0 (-2.0-35.0) | 13.0 (5.0-20.0) | 35.5 (19.3-46.5) | 9.5 (-4.0-34.8) | <0.001^β^ |
| **SF-12 score at baseline,** median (IQR)^µ^ |  |  |  |  |  |
| Physical Component Summary Score | 43.4 (35.2-52.9) | 53.4 (32.3-54.1) | 39.5 (35.2-55.1) | 43.1 (35.0-49.2) | 0.452^$^ |
| Mental Health Component Summary Score | 37.0 (32.8-41.0) | 32.8 (29.8-36.2) | 42.3 (35.3-44.7) | 38.8 (33.1-42.3) | 0.037^$^ |
| **SF-12 score at 6 months,** median (IQR)^µ^ |  |  |  |  |  |
| Physical Component Summary Score | 56.5 (47.3-60.9) | 49.9 (42.7-60.9) | 55.2 (48.9-63.5) | 56.7 (46.6-61.1) | 0.667^$^ |
| Mental Health Component Summary Score | 33.6 (28.9-39.5) | 33.2 (28.9-39.6) | 29.5 (27.5-37.1) | 34.0 (28.6-40.1) | 0.735^$^ |
| **Δ baseline – 6 months scores** |  |  |  |  |  |
| Physical Component Summary Score | 11.6 (3.9-20.1) | 8.7 (-10.7-13.6) | 10.1 (5.2-22.5) | 11.9 (4.0-20.2) | <0.001^β^ |
| Mental Health Component Summary Score | 15.7 (10.3-26.2) | 20.1 (10.4-26.7) | 12.6 (5.6-27.1) | 15.3 (10.3-24.7) | 0.024^β^ |
| ^¥^ Only calculated for patients with complete questionnaires at baseline and 6 months follow-up (n=35).  ^µ^  Only calculated for patients with complete questionnaires at baseline and 6 months follow-up (n=36).^$^ Kruskall-Wallis test was used for non-normal distributed data. ^β^ Wilcoxon signed-rank test. | | | | | |

**Supplementary Table 6 Duration of symptoms and preoperative opioid use on pain relief**

|  | **No complete pain relief** | **Complete pain relief** | ***P*** |
| --- | --- | --- | --- |
| **Duration of symptoms in months**, no. (%) |  |  | 0.037^a^ |
| ≤75^th^ percentile | 49 (56.3) | 38 (43.7) |  |
| > 75^th^ percentile | 21 (80.8) | 5 (19.2) |  |
| **Duration of preoperative opioid use**, no. (%) |  |  | 0.302^a^ |
| Opioid use ≤ 6 months | 24 (64.9) | 13 (35.1) |  |
| Opioid use > 6 months | 28 (77.8) | 8 (38.1) |  |
| 75^th^ percentile of duration of symptoms is 41 months. Duration of symptoms ≤ 75^th^ percentile (n=87); duration of symptoms > 75^th^ percentile (n=26). Opioid use ≤ 6 months (n=37); opioid use > 6 months (n=36). ^a^ Chi-square or Fishers exact test was used for categorical variables | | | |

**Supplementary Table 7 Surgery in patients with solely main pancreatic duct dilation**

|  | **LPJ and Frey**  **(n=37)** | **Other procedures**  **(n=53)** | ***P*** |
| --- | --- | --- | --- |
| **In-patient stay**, days, median (IQR) | 8.0 (6.0-12.0) | 12.0 (7.0-19.0) | 0.006^$^ |
| **Postoperative outcomes, 90 days,** no. (%) |  |  |  |
| Major complications (Clavien-Dindo ≥ 3) | 4 (10.8) | 12 (22.6) | 0.173^a^ |
| Readmission | 7 (18.9) | 7 (13.2) | 0.558^a^ |
| Reintervention | 2 (5.4) | 8 (15.1) | 0.188^a^ |
| Mortality | 0 | 1 (1.9) | >0.999^a^ |
| **Pain outcomes at 6 months**^≠^ |  |  |  |
| Complete pain relief, no. (%) | 10 (38.5) | 9 (37.5) | >0.999^a^ |
| Partial pain relief, no. (%) | 10 (62.5) | 8 (53.3) | 0.772^a^ |
| **Δ baseline – 6 months scores** |  |  |  |
| **VAS**, median (IQR)^≠^ | 55.0 (30.5-75.0) | 50.0 (40.3-76.8) | 0.711^$^ |
| **PANQOLI**, median (IQR)^±^ | 19.0 (7.3-37.0) | 23.0 (7.0-40.5) | 0.935^$^ |
| **SF-12**, median (IQR)^¥^ |  |  |  |
| Physical Component Summary Score | 10.8 (-0.8-30.6) | 14.6 (4.9-22.4) | 0.555^$^ |
| Mental Health Component Summary Score | 12.7 (5.7-26.0) | 25.3 (5.6-23.2) | 0.881^$^ |
| Guideline concordant surgery includes lateral pancreatojejunostomy (LPJ; n=25) and Frey (n=12). Other procedures include Beger and Hamburg procedure, pancreatoduodenectomy, distal pancreatectomy and total pancreatectomy. | | | |

**Supplementary Figure S2 Flowchart of enrollment of study patients**


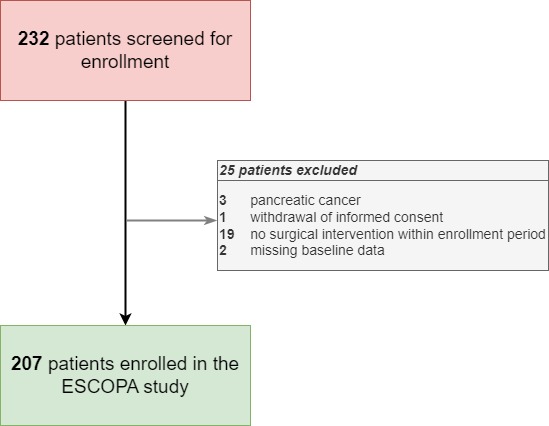

Supplement: znaf068_Supplementary_Data [file znaf068_supplementary_data.docx]
